# Long non-coding RNA NUT family member 2A-antisense RNA 1 sponges microRNA-613 to increase the resistance of gastric cancer cells to matrine through regulating oxidative stress and vascular endothelial growth factor A

**DOI:** 10.18632/aging.204135

**Published:** 2022-06-27

**Authors:** Haifeng Ying, Yinchun Jin, Yuanbiao Guo, Qiong Li, Ming Ruan, Wenhua Zhu, Chen Yang, Qinyu Li, Lan Zheng

**Affiliations:** 1 Ruijin Hospital, Shanghai Jiao Tong University School of Medicine, Shanghai, China

**Keywords:** gastric cancer, matrine, NUT family member 2A-antisense RNA 1, microRNA-613, vascular endothelial growth factor A

## Abstract

Matrine has been shown to play a role in the suppression of gastric cancer (GC) tumorigenesis. However, whether long non-coding RNA NUT family member 2A-antisense RNA 1 (NUTM2A-AS1) is involved in matrine-induced inhibition of GC remains unknown. 3-(4,5-Dimethylthiazol-2-yl)-2,5-diphenyltetrazolium bromide, cell colony formation, and terminal deoxynucleotidyl transferase dUTP nick-end labeling assays were employed to determine the proliferation, viability, and apoptosis of GC cells, respectively. The Cancer Genome Atlas database suggested an association between NUTM2A-AS1 and GC. The reverse transcription-quantitative polymerase chain reaction was used to quantify relative levels of NUTM2A-AS1, miR-613, and vascular endothelial growth factor A (VEGFA). Reactive oxygen species generation, glutathione content, and superoxide dismutase activity were determined by corresponding reagents or assay kits. NUTM2A-AS1 knockdown led to attenuated cell viability and proliferation, as well as to enhanced apoptosis of N87 and AGS cells treated with matrine. These changes were prevented by an inhibitor of microRNA (miR)-613. Importantly, NUTM2A-AS1 expression was positively associated with tumor progression in patients with GC. NUTM2A-AS1 and miR-613 regulated the generation of reactive oxygen species, the content of glutathione, and the activity of superoxide dismutase. VEGFA served as an important effector for the NUTM2A-AS1/miR-613-regulated resistance of GC cells to matrine. These results reveal a novel mechanism of matrine resistance in GC.

## INTRODUCTION

Gastric cancer (GC) has been ranked as the second most common cause of cancer-associated mortality in Eastern Asia, particularly in China [1, 2]. Surgical resection is the most effective treatment strategy for patients with early-stage GC [3]. When GC progresses to an advanced stage (unresectable or metastatic), chemotherapy remains the major approach to treatment [4]. As drug resistance commonly occurs, it is urgent to identify novel biomarkers or targets for developing new therapies for GC.

Matrine is one of the bioactive components derived from *Sophora flavescens* [5]. Traditionally, matrine has been used for the treatment of cancer, arrhythmias, skin diseases, and hepatitis in China [6, 7]. Previous studies have shown that matrine can suppress the proliferation of tumor cells. For example, matrine was shown to inhibit melanoma and cervical cancer tumorigenesis [8, 9]. Notably, matrine was also found to induce GC cell death by regulating various downstream targets [10].

Long non-coding RNAs (lncRNAs) are defined as ~200-nt RNAs without protein translation ability [11, 12]. Previous studies have shown that lncRNAs are implicated in various biological processes, including tumorigenesis and drug resistance [13, 14]. Recently, it has been reported that NUT family member 2A-antisense RNA 1 (NUTM2A-AS1) is highly expressed in non-small cell lung cancer and plays a role in GC [15, 16].

MicroRNAs (miRNAs or miRs) are small non-coding RNAs of ~22 nt in length that bind to the 3′-untranslated region of mRNAs of target genes to regulate their expression [17]. It has been shown that miRNAs are involved in a variety of cancer types, including osteosarcoma and colorectal cancer [18, 19]. LncRNAs act as competitive endogenous RNAs to interact with miRNAs via miRNA recognition elements that sequester the miRNA-RNA-induced silencing complex away from downstream genes [20]. Consequently, lncRNAs might exert their effects via miRNAs during cancer progression. In previous studies, miR-613 was shown to suppress tumor cell proliferation and migration, and it was able to target SRY-box transcription factor 9, 6-phosphofructo-2-kinase/fructose-2,6-biphosphatase 2, and cyclin-dependent kinase 9 to inhibit the tumorigenic potential of GC cells [21–23].

Vascular endothelial growth factor A (VEGFA) plays a role in tumor growth and metastasis. In previous studies, VEGFA was shown to be regulated by various miRNAs including miR-150-5p [24], miR-130b [25], and miR-5047 [26]. Thus, it is common to link VEGFA and miRNAs in tumor progression.

The purpose of the present study was to elucidate the molecular mechanism underlying GC progression. We hope that the findings will broaden our current insights into lncRNA-mediated GC progression and facilitate the development of novel therapeutics for GC.

## RESULTS

### The loss of NUTM2A-AS1 attenuates GC tumorigenesis following matrine treatment

**Figure 1:**
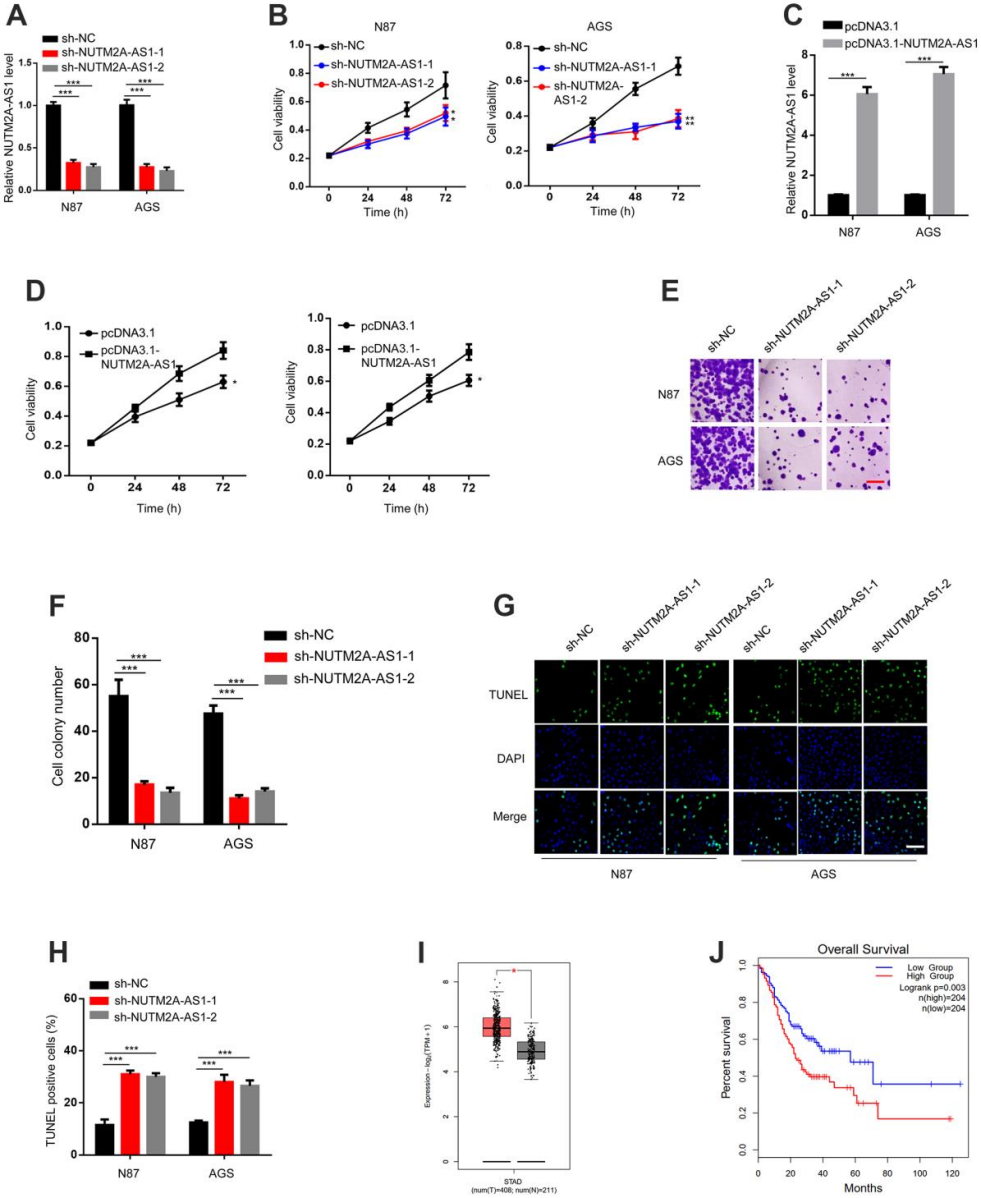
**The loss of NUTM2A-AS1 attenuates GC tumorigenesis following matrine treatment.** (**A**) The RT-qPCR was employed to evaluate the levels of NUTM2A-AS1 in N87 and AGS cells transfected with sh-NC, sh-NUTM2A-AS1-1, or sh-NUTM2A-AS1-2. ^***^P < 0.001. (**B**) The viability of N87 or AGS cells transfected with sh-NC, sh-NUTM2A-AS1-1, or sh-NUTM2A-AS1-2 following matrine treatment was examined by using the MTT assay. ^*^P < 0.05, ^**^P < 0.01. (**C**) The RT-qPCR was employed to evaluate the levels of NUTM2A-AS1 in N87 and AGS cells transfected with pcDNA3.1 or pcDNA3.1-NUTM2A-AS1. ^***^P < 0.001. (**D**) The viability of N87 or AGS cells transfected with pcDNA3.1 or pcDNA3.1-NUTM2A-AS1 following matrine treatment was examined by using the MTT assay. ^*^P < 0.05. (**E**, **F**) Cell colonies of N87 and AGS cells transfected with sh-NC, sh-NUTM2A-AS1-1, or sh-NUTM2A-AS1-2 following matrine treatment. Scale bar, 5 μm. ^***^P<0.001. (**G**, **H**) Apoptosis of N87 and AGS cells transfected with sh-NC, sh-NUTM2A-AS1-1, or sh-NUTM2A-AS1-2 following matrine treatment was examined by using the TUNEL assay. Scale bar, 5 μm. ^***^P < 0.001. (**I**) The Cancer Genome Atlas analysis of patients with GC showing the NUTM2A-AS1 levels in normal tissues and tumors. ^*^P < 0.05. (**J**) Kaplan-Meier plot to evaluate the overall survival of patients with GC and high or low NUTM2A-AS1 levels. P = 0.003, high (n = 204), low (n = 204). RT-qPCR, reverse transcription-quantitative polymerase chain reaction; NUTM2A, NUT family member 2A; AS1, antisense RNA 1; GC, gastric cancer; sh, small hairpin; NC, negative control.

To investigate the role of NUTM2A-AS1 in matrine-regulated GC tumorigenesis, NUTM2A-AS1 knockdown was performed by using two small hairpin (sh)RNAs. The reverse transcription-quantitative polymerase chain reaction (RT-qPCR) results demonstrated that both sh-NUTM2A-AS1-1 and sh-NUTM2A-AS1-2 decreased the expression of NUTM2A-AS1 compared with that caused by sh-negative control (NC) (Figure 1A).

NUTM2A-AS1-knockdown N87 and AGS cells showed reduced viability and cell colony numbers following matrine treatment (Figure 1B, 1E, 1F). In contrast, NUTM2A-AS1-overexpressing N87 and AGS cells showed enhanced viability following matrine treatment (Figure 1C, 1D). NUTM2A-AS1 knockdown led to increased cell apoptosis (Figure 1G, 1H). Taken together, these findings demonstrated that NUTM2A-AS1 contributed to the effect of matrine on GC tumorigenesis.

Next, we determined whether NUTM2A-AS1 was involved in the overall survival of patients with GC. By analyzing The Cancer Genome Atlas (TCGA) datasets, upregulated NUTM2A-AS1 expression was observed in tumor compared with non-tumor tissues (Figure 1I). Importantly, a low NUTM2A-AS1 level was associated with increased overall survival time (Figure 1J).

### MiR-613 prevents NUTM2A-AS1-induced resistance of GC cells to matrine

**Figure 2:**
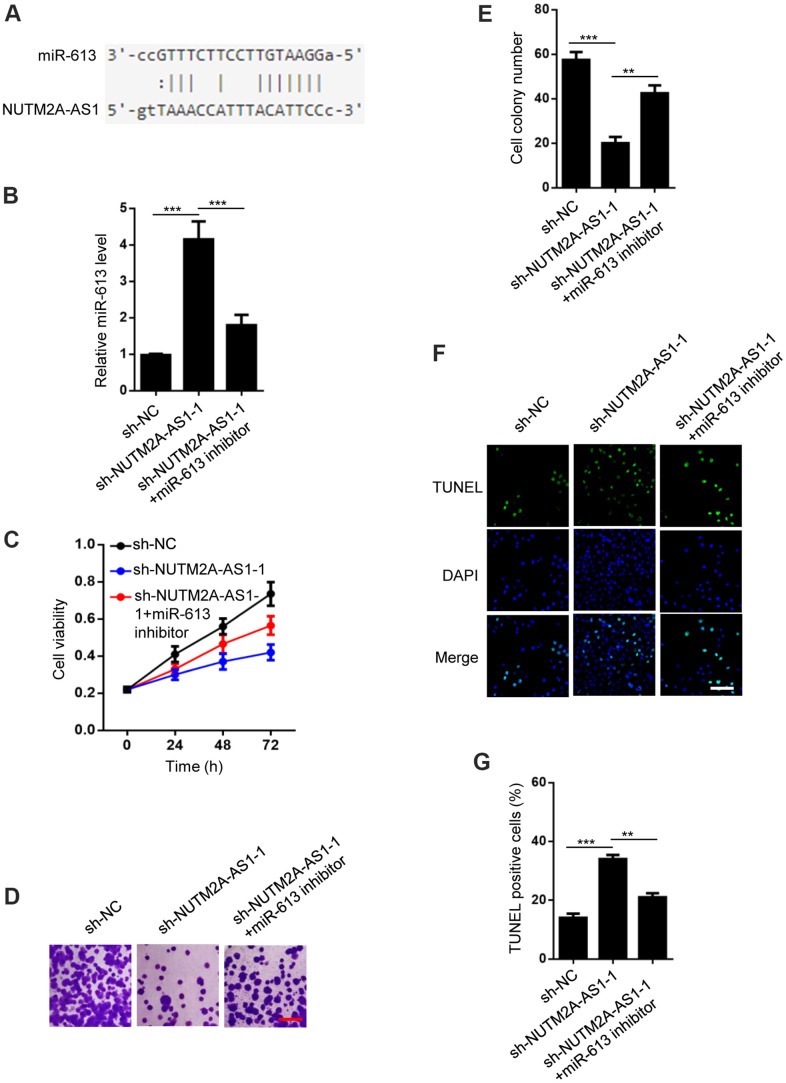
**MiR-613 rescues NUTM2A-AS1-regulated matrine resistance in GC cells.** (**A**) StarBase 2.0 shows that miR-613 is a target of NUTM2A-AS1. (**B**) The reverse transcription-quantitative polymerase chain reaction was employed to evaluate the levels of miR-613 in N87 cells transfected with sh-NC, sh-NUTM2A-AS1-1, or sh-NUTM2A-AS1-1 plus the miR-613 inhibitor. ^***^P < 0.001. (**C**) The viability of N87 cells transfected with sh-NC, sh-NUTM2A-AS1-1, or sh-NUTM2A-AS1-1 plus the miR-613 inhibitor under matrine treatment was examined by using the MTT assay. ^*^P < 0.05, ^**^P < 0.01. (**D**, **E**) Cell colonies of N87 cells transfected with sh-NC, sh-NUTM2A-AS1-1, or sh-NUTM2A-AS1-1 plus the miR-613 inhibitor under matrine treatment. Scale bar, 5 μm. ^**^P < 0.01, ^***^P < 0.001. (**F**, **G**) Apoptosis of N87 cells transfected with sh-NC, sh-NUTM2A-AS1-1, or sh-NUTM2A-AS1-1 plus the miR-613 inhibitor under matrine treatment was examined by using the TUNEL assay. Scale bar, 5 μm. ^**^P < 0.01, ^***^P < 0.001. DAPI, 4',6-diamidino-2-phenylindole; NUTM2A, NUT family member 2A; AS1, antisense RNA 1; GC, gastric cancer; miR, microRNA; sh, small hairpin; NC, negative control; TUNEL, terminal deoxynucleotidyl transferase dUTP nick-end labeling.

To determine the downstream miRNAs responsible for NUTM2A-AS1-mediated matrine resistance in GC cells, starBase v2.0 was used to identify miR-613 as a potential target of NUTM2A-AS1 (Figure 2A). NUTM2A-AS1 knockdown resulted in increased expression of miR-613 (Figure 2B). 3-(4,5-Dimethylthiazol-2-yl)-2,5-diphenyltetrazolium bromide (MTT) and cell colony formation assays demonstrated that NUTM2A-AS1 depletion impaired N87 cell viability and proliferation, respectively, following treatment with matrine. The miR-613 inhibitor rescued the impaired cell viability and proliferation (Figure 2C–2E). NUTM2A-AS1 depletion caused enhanced apoptosis of N87 cells under matrine treatment, which was rescued by the miR-613 inhibitor (Figure 2F, 2G). These findings indicate that miR-613 was critical for NUTM2A-AS1-regulated resistance of GC cells to matrine.

### NUTM2A-AS1 and miR-613 regulate oxidative stress signaling

**Figure 3:**
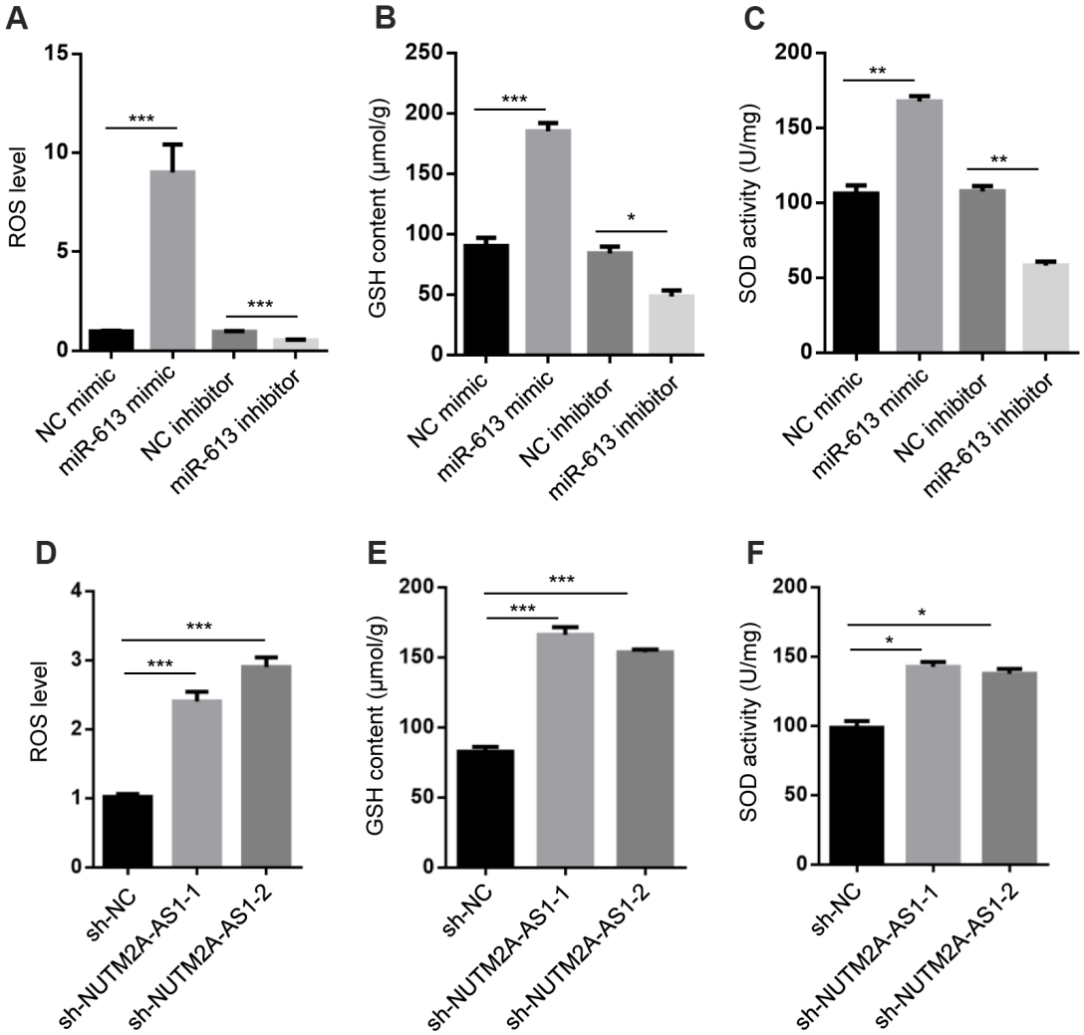
**NUTM2A-AS1 and miR-613 regulate oxidative stress signaling.** (**A**) ROS levels were measured in N87 cells transfected with the NC mimic, miR-613 mimic, NC inhibitor, or miR-613 inhibitor. ^***^P < 0.001. (**B**) GSH contents were measured in N87 cells transfected with the NC mimic, miR-613 mimic, NC inhibitor, or miR-613 inhibitor. ^*^P < 0.05, ^***^P < 0.001. (**C**) SOD activity was measured in N87 cells transfected with the NC mimic, miR-613 mimic, NC inhibitor, or miR-613 inhibitor. ^**^P < 0.01. (**D**) ROS levels were measured in N87 cells transfected with sh-NC, sh-NUTM2A-AS1-1, or sh-NUTM2A-AS1-2. ***P < 0.001. (**E**) GSH contents were measured in N87 cells transfected with sh-NC, sh-NUTM2A-AS1-1, or sh-NUTM2A-AS1-2. ^***^P < 0.001. (**F**) SOD activity was measured in N87 cells transfected with sh-NC, sh-NUTM2A-AS1-1, or sh-NUTM2A-AS1-2. ^*^P < 0.05. NUTM2A, NUT family member 2A; AS1, antisense RNA 1; miR, microRNA; sh, small hairpin; NC, negative control; ROS, reactive oxygen species; GSH, glutathione; SOD, superoxide dismutase.

A previous report showed that miR-613 modulated reactive oxygen species (ROS) in neuronal cells [27]. Based on this observation, we hypothesized that NUTM2A-AS1 and miR-613 may regulate oxidative stress signaling. The miR-613 mimic promoted ROS generation, and increased the glutathione (GSH) level and superoxide dismutase (SOD) activity, while the miR-613 inhibitor decreased ROS generation, the GSH level, and SOD activity in N87 cells treated with matrine (Figure 3A–3C). In addition, NUTM2A-AS1 knockdown increased ROS generation, the GSH level, and SOD activity in N87 cells treated with matrine (Figure 3D–3F). Taken together, these findings demonstrated that NUTM2A-AS1 and miR-613 regulated oxidative stress signaling in GC cells.

### VEGFA is critical for miR-613-mediated suppression of GC in response to matrine treatment

**Figure 4:**
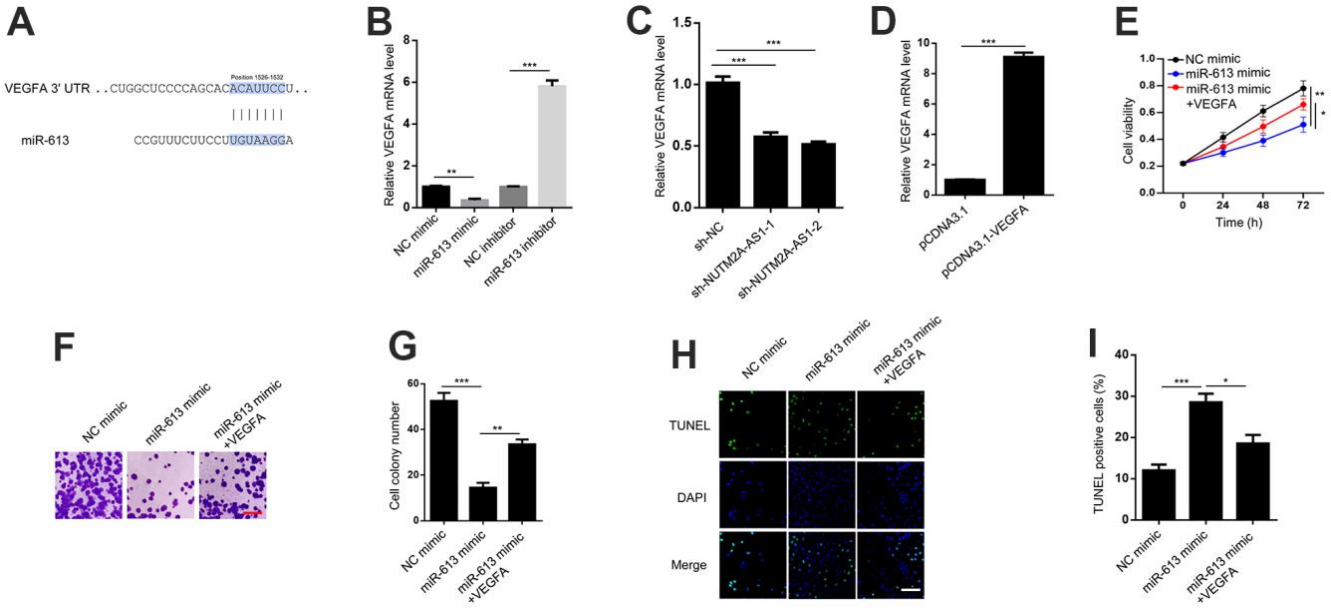
**VEGFA is critical for miR-613-mediated suppression of gastric cancer under matrine treatment.** (**A**) TargetScan shows that VEGFA is a potential target of miR-613. (**B**) VEGFA mRNA levels were detected by the RT-qPCR in N87 cells transfected with the NC mimic, miR-613 mimic, NC inhibitor, or miR-613 inhibitor. ^**^P < 0.01, ^***^P < 0.001. (**C**) VEGFA mRNA levels were detected by the RT-qPCR in N87 cells transfected with sh-NC, sh-NUTM2A-AS1-1 or sh-NUTM2A-AS1-2. ^***^P < 0.001. (**D**) VEGFA expression levels were determined by the RT-qPCR in N87 cells transfected with pcDNA3.1 or pcDNA3.1-VEGFA. ^***^P < 0.001. (**E**) The MTT assay was used to evaluate the viability of N87 cells transfected with the NC mimic, miR-613 mimic, or miR-613 mimic plus VEGFA under matrine treatment. ^*^P < 0.05, ^**^P < 0.01. (**F**, **G**) Cell colonies of N87 cells transfected with the NC mimic, miR-613 mimic, or miR-613 mimic plus VEGFA under matrine treatment. Scale bar, 5 μm. ^**^P < 0.01, ^***^P < 0.001. (**H**, **I**) The apoptosis of N87 cells transfected with the NC mimic, miR-613 mimic, or miR-613 mimic plus VEGFA under matrine treatment was examined by the TUNEL assay. Scale bar, 5 μm. ^*^P < 0.05, ^***^P < 0.001. VEGFA, vascular endothelial growth factor A; miR, microRNA; RT-qPCR, reverse transcription-quantitative polymerase chain reaction; NC, negative control; TUNEL, terminal deoxynucleotidyl transferase dUTP nick-end labeling.

TargetScan software was used to predict the potential downstream effector of NUTM2A-AS1/miR-613 in GC (Figure 4A). The RT-qPCR results revealed that the miR-613 mimic suppressed, whereas the miR-613 inhibitor promoted, VEGFA expression (Figure 4B). These data were in agreement with previous findings [19, 28]. In addition, NUTM2A-AS1 knockdown attenuated the VEGFA expression level (Figure 4C).

To elucidate whether VEGFA was critical for miR-613-modulated GC tumorigenesis, VEGFA was overexpressed in N87 cells (Figure 4D). The MTT and cell colony formation assays revealed that VEGFA could rescue miR-613-inhibited cell viability and proliferation following matrine treatment (Figure 4E–4G). In addition, VEGFA attenuated miR-613-enhanced cell apoptosis following matrine treatment (Figure 4H, 4I). In summary, VEGFA acted as key effector of miR-613-induced suppression of GC under matrine treatment.

## DISCUSSION

During GC treatment worldwide, chemotherapy resistance is a common problem [29]. Thus, novel agents, including natural compounds, must be developed for treating GC. Matrine is a component of traditional Chinese medicine. Previous studies showed that matrine inhibited GC and hepatocellular carcinoma progression by stimulating cell apoptosis [30, 31]. In agreement with published data, the present *in vitro* results demonstrated that matrine attenuated cell viability and proliferation, and enhanced cell apoptosis.

Non-coding RNAs include lncRNAs, miRNAs, circular RNAs, and small nucleolar RNAs, among others, and have been reported to be involved in diverse biological processes [32]. The present study explored the potential role of lncRNA NUTM2A-AS1 in GC tumorigenesis. NUTM2A-AS1 knockdown markedly reduced GC cell viability and proliferation, and promoted cell apoptosis. In patients with GC, tumors expressed more NUTM2A-AS1 than normal tissue, and a lower level of tumor NUTM2A-AS1 was associated with prolonged survival time.

ROS are generated naturally by cellular metabolism, as well as by some xenobiotics [33]. Intracellular ROS derived from chemotherapeutic agents can kill tumor cells [34]. It has been suggested that ROS could induce GC cell apoptosis and drug sensitivity [35–37]. In the present study, it was observed that NUTM2A-AS1 and miR-613 regulated ROS production in N87 cells. These findings led us to hypothesize that ROS may be a key effector for NUTM2A-AS1/miR-613-mediated matrine-resistant GC.

VEGF has been proposed to serve as a crucial gene for promoting angiogenesis during tumor metastasis [38]. VEGFA overexpression is found in various tumors, including GC [39]. The present study confirmed that VEGFA acted as a downstream effector of miR-613 in N87 cells. The mechanism by which VEGFA influences GC tumorigenesis remains unknown. According to previous research, VEGFA could activate the phosphoinositide 3-kinase/AKT and extracellular signal-regulated kinase 1/2 signaling pathways, which may be potential mechanisms for NUTM2A-AS1/VEGFA-induced GC tumorigenesis [40]. In addition, it is interesting that oxidative stress can regulate VEGFA gene transcription [41]. This observation led us to perform the current study that clarifies the relationship between NUTM2A-AS1, miR-613, ROS, and VEGFA.

## CONCLUSIONS

The NUTM2A-AS1/miR-613/ROS/VEGFA axis is important for the inhibition of GC progression by matrine. The present findings enrich our understanding of GC treatment with matrine, and may facilitate the development of novel therapeutics for GC by targeting the NUTM2A-AS1/miR-613/ROS/VEGFA axis. However, the role of this axis in GC presented in this study was limited to the cellular and molecular biology levels. Thus, additional efforts are needed to confirm this finding at the animal level and, ultimately, in human clinical trials.

## MATERIALS AND METHODS

### Cell culture

Human GC cells (N87 and AGS) and 293T cells were obtained from the The Cell Bank of Type Culture Collection of The Chinese Academy of Sciences (Shanghai, China). Cells were cultured in Dulbecco’s Modified Eagle’s Medium (Gibco, Gaithersburg, MD, USA) supplemented with 10% fetal bovine serum (Gibco) and 1% penicillin/streptomycin at 37° C in the presence of 5% CO_2_.

### Transfection

VEGFA and NUTM2A-AS1 were amplified and cloned into the pcDNA3.1 vector. pcDNA3.1-VEGFA or pcDNA3.1-NUTM2A-AS1 was then transiently transfected into N87 or AGS cells using Lipofectamine^®^ 2000 (Invitrogen). After 48 h of transfection, the cell lysates were collected and used for subsequent experiments.

### Stable cell line generation

To generate stable cell lines, ~2 μg of the vectors [1 μg pLKO.1-shRNA, 0.5 μg pVSVG, and 0.5 μg of pPAX2] were transfected into 293T cells by using Lipofectamine^®^ 2000. After 36 h, the lentiviruses were harvested and stored at -80° C. Next, the viruses were used to infect N87 and AGS cells. To obtain resistant cells, ~1 μg/mL puromycin was used. The shRNA sequences were as follows: sh-NC, 5′-AUCGGCAACUAGGCAUCAUCAG-3′; sh-NUTM2A-AS1-1, 5′-GGGACAGUGUAUGCAAGAA-3′; and sh-NUTM2A-AS1-2, 5′-GGACAGUGUAUGCAAGAAU-3′.

### MTT assay

N87 and AGS cells were seeded in 96-well plates (~5,000 cells/well). MTT reagent (Sigma-Aldrich, St. Louis, MO, USA) was added and the cells were incubated for 4 h at 37° C in the presence of 5% CO_2_. At different time points (0, 24, 48, and 72 h), 150 μL dimethyl sulfoxide were added to each well and incubated with the cells for 10 min. Finally, the optical density at 490 nm was examined.

### Colony formation assay

N87 and AGS cells (2 × 10^4^ cells) were seeded in 6-well plates and cultured for 2 weeks. The cell colonies were fixed with 4% paraformaldehyde. Crystal violet (0.005%) was used to stain the colonies, and the number was counted under a light microscope.

### Terminal deoxynucleotidyl transferase dUTP nick-end labeling (TUNEL) assay

Transfected N87 and AGS cells were seeded in each well of a 24-well plate. TUNEL signals were detected according to the manufacturer’s instructions (Beyotime Institute of Biotechnology, Haimen, China). The cell nuclei were stained with 4',6-diamidino-2-phenylindole.

### TCGA analysis

TCGA datasets of patients with GC were downloaded and analyzed (408 tumor and 211 normal tissues). The relative expression of NUTM2A-AS1, overall survival, and stage were analyzed. A Kaplan-Meier plot was used to evaluate the overall survival of patients.

### ROS determination

GC cells were incubated with 10 μM 2,7-dichlorodi-hydrofluorescin diacetate at 37° C for 30 min to determine ROS generation. The activity of SOD and content of GSH were measured with cell lysates using appropriate kits (Construction Bioengineering Research, Nanjing, China).

### RT-qPCR

Total RNA was extracted from GC cells using TRIzol^®^ (Invitrogen) according to the manufacturer’s instructions. The RNA concentration was determined using a NanoDrop instrument (NanoDrop Technologies, Wilmington, DE, USA). For RT-qPCR, a PrimeScript RT Reagent Kit (Takara Bio, Dailan, China) was used to generate cDNA with 1 μg total RNA. RT-qPCR was performed with the SYBR Green I Master Mix Kit. β-Actin or U6 acted as an internal control. Relative gene levels were calculated by using the 2^-ΔΔCq^ method. The primers used were as follows: NUTM2A-AS1: forward, 5′-CTCGACTCAGTCCTCCAGC-3′ and reverse, 5′-GCCTCCTCCTCTTGCTTCAT-3′; miR-613: forward, 5′-GTGAGTGCGTTTCCAAGTGT-3′ and reverse, 5′-GGGTCCCTTCACACTTGGAA-3′; VEGFA: forward, 5′-ATCCAATCGAGACCCTGGTG-3′ and reverse, 5′-ATCTCTCCTATGTGCTGGCC-3′; β-actin: forward, 5′-GGGAAATCGTGCGTGACATT-3′ and reverse, 5′-AGGTAGTTTCGTGGATGCCA-3′; and U6: forward, 5′- CGCTTCGGCAGCACATATACTA-3′ and reverse, 5′- GAATTTGCGTGTCATCCTTGCG-3′.

### Statistical analyses

Statistical analyses were performed with Prism 8.0 software (GraphPad Software, La Jolla, CA, USA). Two groups were compared by using the unpaired Student’s t-test, while multiple groups were compared by a one-way analysis of variance followed by Tukey’s post-hoc test. P < 0.05 was considered to indicate a statistically significant difference.

### Data availability statement

The data supporting this research are available from the corresponding author upon reasonable request.
